# The prognostic value of tumour–stroma ratio in primary breast cancer with special attention to triple-negative tumours: a review

**DOI:** 10.1007/s10549-018-4987-4

**Published:** 2018-10-09

**Authors:** C. J. H. Kramer, K. M. H. Vangangelt, G. W. van Pelt, T. J. A. Dekker, R. A. E. M. Tollenaar, W. E. Mesker

**Affiliations:** Department of Surgery, Leiden University Medical Center, Albinusdreef 2, 2333 ZA Leiden, The Netherlands

**Keywords:** Breast cancer, Triple-negative breast cancer, Tumour–stroma ratio, Prognosis, Review

## Abstract

**Purpose:**

There is a strong need to improve the prognostication of breast cancer patients in order to prevent over- and undertreatment, especially when considering adjuvant chemotherapy. Tumour stroma characteristics might be valuable in predicting disease progression.

**Methods:**

Studies regarding the prognostic value of tumour–stroma ratio (TSR) in breast cancer are evaluated.

**Results:**

A high stromal content is related to a relatively poor prognosis. The most pronounced prognostic effect of this parameter seems to be observed in the triple-negative breast cancer (TNBC) subtype.

**Conclusions:**

TSR assessment might represent a simple, fast and reproducible prognostic factor at no extra costs, and could possibly be incorporated into routine pathological diagnostics. Despite these advantages, a robust clinical validation of this parameter has yet to be established in prospective studies.

**Electronic supplementary material:**

The online version of this article (10.1007/s10549-018-4987-4) contains supplementary material, which is available to authorized users.

## Introduction

According to the European cancer statistics for 2018, the estimated number of new breast cancer cases is 522.500 and the estimated number of deaths is 137.700 [1]. Breast tumours are classified in four molecular subtypes, namely luminal A, luminal B, human epidermal growth factor receptor 2 (HER2)-enriched and basal-like [2, 3]. The triple-negative breast cancer (TNBC) belongs to the basal-like phenotype in the vast majority, which is an aggressive form of breast cancer with a shorter relapse-free period (RFP) and relative survival compared to luminal A and B [4, 5]. However, gene-expression analyses have shown that this group is notoriously heterogeneous, with some molecular subtypes even associated with a relatively favourable prognosis [5]. Approximately 16% of all breast cancer cases are represented by TNBC [6].

In recent years, extensive research has been performed to discover new prognostic biomarkers and determine optimal prognostication schemes for breast cancer patients. Molecular tests, such as the 70-gene signature (MammaPrint, Agenda BV, The Netherlands) and the 21-gene assay (Oncotype DX, Genomic Health, United States), have shown to improve clinical decision making in early-stage breast cancer of certain molecular and clinical subtypes, such as oestrogen-positive (ER+) or HER2-negative (HER2−) breast cancer [7, 8]. These molecular markers are now endorsed into routine clinical practice, according to the American Society of Clinical Oncology Clinical Practice guideline, in order to reduce the administration of adjuvant chemotherapy and prevent overtreatment [9].

Despite the fact that alterations in the tumour microenvironment have been recognised as important drivers of tumour progression, the tumour environment has not been integrated in routine clinical decision making yet. A parameter which translates the amount of tumour-associated stroma is the tumour–stroma ratio (TSR), which has been extensively described as a rich source of prognostic information for various solid cancer types [10–38]. The TSR was first described as a prognostic factor in breast cancer in 2011 by De Kruijf et al. and has been validated in numerous studies [12–15, 17].

For the TSR assessment, the amount of tumour-associated stroma is determined on routine haematoxylin and eosin (H&E) stained slides of the primary tumour tissue. Each tumour is assigned to either the stroma-high or stroma-low category based on a set cut-off value [10].

In this review, literature investigating the effect of the TSR as a prognostic factor in female breast cancer is discussed with a special interest in the prognostic effect in TNBC patients.

## Rationale

The influence of the tumour-associated stroma on epithelial tumour progression is mostly derived from functional in vitro studies. Similarly, those in vitro studies have demonstrated events in the stromal compartment that occur during carcinogenesis and could contribute to tumour progression. The production of growth factors and proteases by cancer cells initiate changes in the stromal environment [39]. Those alterations lie within remodelling of the matrix, recruitment of fibroblasts, the migration of immune cells and angiogenesis, all contributing to tumour progression [40]. Cancer-associated fibroblasts (CAFs) contribute to carcinogenesis through the development of unique functions, including an amplified extracellular matrix (ECM) production, higher proliferation rate and the secretion of several cytokines, like vascular endothelial growth factor (VEGF), stromal cell-derived factor 1 (SDF1) and platelet-derived growth factor (PDGF), leading to angiogenesis [40]. Transforming growth factor-β (TGF-β) is another factor that is thought to be strongly involved in the tumour-promoting effects of CAFs as described in colon cancer by Hawinkels et al. [41]. Those behavioural modifications lead to an elevated expression of enzymes, like matrix metalloproteinases (MMPs), resulting in remodelling and deposition of the ECM, with concurrently the release of pro-angiogenic factors [42].

The ECM is frequently disorganised in tumours. One of the most important mechanisms in the ECM contributing to tumour progression is collagen crosslinking. Due to crosslinking collagen by lysyl oxidase (LOX), the ECM of the tumour becomes more stiff, leading to increased focal adhesions and enhanced PI3K signalling, thereby indirectly ensure tumour progression [43]. Besides the fact that alterations in the tumour niche lead to progression directly, the tumourigenesis can also be strengthened indirectly due to the aforementioned production of pro-angiogenic factors by CAFs and immune cells. Thus, during the process of tumourigenesis changes occur in the organisation of stromal cells, contributing both directly and indirectly to tumour growth and progression.

Previous studies investigating gene-expression profiles in stromal cells have demonstrated gene signatures related to clinical outcome and response to treatment in breast cancer [44, 45]. Clinical application of these signatures was impractical and a definitive indication was never discovered. These studies did provide a strong indication that valuable clinical information was ignored by solely focusing on the epithelial compartment. As the stromal processes that are reflected by these assays likely have a quantitative relationship with the amounts of stromal tissue within the tumour, quantitative stromal parameters might equally express prognostic information just by morphology alone [45].

## Methods used for TSR assessment

In literature, two methods are described for TSR assessment in breast cancer: visual scoring, utilised by Mesker et al.; and automated point counting, a semi-automated approach, utilised by West et al. [10, 18].

### Visual eyeballing

**Fig. 1:**
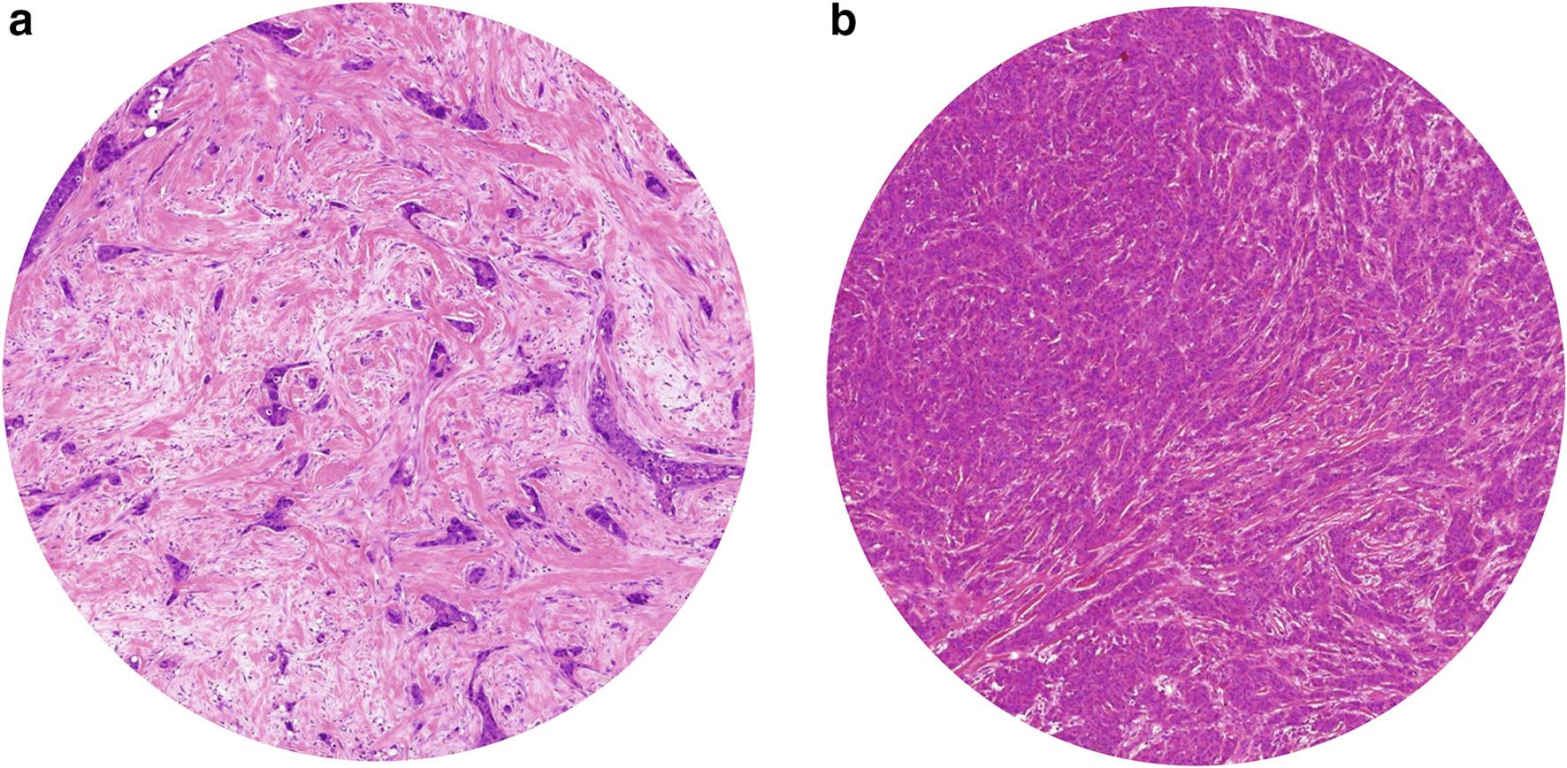
Microscopic evaluation of tumour–stroma ratio on hematoxylin and eosin stained sections of breast tumours with a 10 × objective categorised in stroma-high tumours (> 50% stroma) and stroma-low tumours (≤ 50% stroma) by visual eyeballing. **a** Stroma-high **b** Stroma-low

Mesker et al. and others determined TSR by visual eyeballing [10, 12–17]. This microscopic determination of the amount of stroma in the primary tumour is performed on routine H&E stained slides. A 2.5 × or 5 × objective is used to determine the most stroma–abundant area on the slide. In this area, image fields with tumour cells at all borders of the image are used to determine the amount of stroma, using a 10 × objective. The stroma percentage is estimated in increments of 10% per image field, considering the highest scored stroma percentage as decisive. Stroma percentage ≤ 50% is categorised as stroma-low and a stroma percentage > 50% is categorised as stroma-high, based on statistical determination, initially performed on colon cancer and subsequently verified for breast cancer (Fig. 1) [10, 18]. Considerable segments of necrosis or *in situ* tumours were excluded in the evaluation of the TSR by neglecting them in the analysis [12, 14].

### Semi-automated point counting

West and colleagues have objectified the measurement by evaluating the tumour tissue slides in colon carcinoma, using 300 random measurement points, validated for breast cancer by Downey et al. [18, 46, 47]. Four-micrometer-thick H&E stained sections are scanned, using a 20 × objective and subsequently two areas without large segments of necrosis are selected with a digital slide viewer. In this method, the two sampled 9 mm^2^ areas are in the tumour-leading edge, as well as in the non-leading edge. The group utilises a grid with a sample of 300 random points, superimposed on the selected area. Under each of the 300 points, the histopathology is categorised in ‘tumour’, ‘stroma’ or ‘unclassified’ (necrosis, blood vessels, inflammation etc.). The ultimate TSR is the proportion of ‘stroma’ under the 300 points, compared with all points per section. In other words, the TSR is the number of points, categorised as ‘stroma’ divided by the total number of points, categorised as ‘tumour’ and ‘unclassified’ [18, 46, 47]. Downey et al. have used 0.49 (i.e. 49%) as a cut-off value in their study in 2014, with ≥ 0.49 being stroma-high and < 0.49 stroma-low, based on statistical analysis [46]. However, in another study, cut-off values of 0.31 TSR for OS and 0.46 for DFS are used [47].

The inter-observer variation of these two methods, determined by the Cohen’s kappa coefficient (Κ) or intraclass correlation coefficient (ICC), lies in the range of 0.68–0.85, indicating substantial to good agreement between observers in both methods (Table 1).

**Table 1:** Detailed overview of studies on the prognostic value of tumour–stroma ratio (TSR) in the entire study population and of triple-negative breast cancer patients (TNBC)

		Author, date	Sample size	Percentage of stroma-high tumours (%)	Population	Method	Inter-observer variation	Outcome (HR (95% CI), *P*-value)	Most favourable prognosis
Entire study population	General BC	De Kruijf et al. [12]	574	68	pT1–4, pNneg–pos, grade I–III	VS	*K* = 0.85	OS: 1.50 (1.18–1.91), *P* = 0.001 RFP: 1.97 (1.47–2.64), *P* < 0.00	Stroma-low
		Dekker et al. [14]	403	40	T1–3, N0–2, grade I–III	VS	*K* = 0.804	OS: 1.60 (1.00-2.57), *P* = 0.050 DFS: 1.85 (1.33–2.59), *P* < 0.001	Stroma-low
		Roeke et al. [17]	737	38	T1–3, Nneg–pos, grade I–III	VS	*K* = 0.68	OS: 1.56 (1.18–2.05), *P* = 0.002 RFS: 1.35 (1.01–1.81), *P* = 0.046 DMFS: 1.52 (1.12–2.06), *P* = 0.008	Stroma-low
	IC NST BC	Gujam et al. [15]	361	30	T1–3, N0– >3, grade I–III	VS	*ICC* = 0.83	CSS: 2.12 (1.37–3.29), *P* = 0.001	Stroma-low
	ER + BC	Downey et al. [46]	118		N0–3, grade I–III	APC	*K* = 0.70	OS: 0.2–0.7, *P* = 0.008 RFS: 0.1–0.6, *P* = 0.006	Stroma-high
	Inflam. BC	Downey et al. [47]	45		N0–3, grade I–III	APC	NM	OS: *P* = 0.53 DFS: *P* = 0.66	No difference
Entire study population	TNBC	Moorman et al. [13]	124	40	pT1–4, pN0–3, grade I–III	VS	*K* = 0.74	OS: 3.00 (1.08–8.32), *P* = 0.034 RFP: 2.39 (1.07–5.29), *P* = 0.033	Stroma-low
Subgroup of main study population	TNBC	De Kruijf et al. [12]	82	56	pT1–4, pNneg-pos, grade I–III	VS		OS: 1.87 (1.07–3.26), *P* = 0.028 RFP: 2.92 (1.36–6.32), *P* = 0.006	Stroma-low
		Dekker et al. [14]	69			VS		DFS: 2.71 (1.11–6.61), *P* = 0.028	Stroma-low
		Roeke et al. [17]	77	26		VS		OS: *P* = 0.221	No difference
	IC NST TNBC	Gujam et al. [15]	151	24	T1–3, N0– >3, grade I–III	VS		CSS: *P* = 0.151	No difference

*BC* breast cancer, *IC NST* invasive carcinoma of no special type, *TNBC* triple-negative breast cancer, *ER+* oestrogen-receptor positive, *inflam*. inflammatory, *VS* visual scoring, *APC* automated point counting, *K* Cohen’s kappa value, *NM* not mentioned, *HR* hazard ratio, *CI* confidence interval, *ICC* intraclass correlation coefficient, *OS* overall survival, *RFP* relapse-free period, *DFS* disease-free survival, *DM* distant metastasis, *CSS* cancer-specific survival, *RFS* recurrence-free survival, *DMFS* distant metastasis-free survival, *Neg* negative, *Pos* positive

## Tumour–stroma ratio in breast cancer patients

The first study on TSR in breast cancer was published by De Kruijf et al. [12]. TSR was estimated by visual eyeballing according to the method described by Mesker et al. [10]. The authors showed that TSR was an independent prognostic parameter in 574 breast cancer patients with invasive breast tumours without distant metastasis (pT1–4, pN0–3, M0). Stroma-high tumours were associated with a worse RFP (Hazard Ratio (HR) 1.97, 95% Confidence Interval (CI) 1.47–2.64, *P* < 0.001) and overall survival (OS) (HR 1.50, 95% CI 1.18–1.91, *P* = 0.001) analysed with multivariate Cox regression analysis (Table 1) [12]. Vangangelt et al. analysed the prognostic value of TSR in a subset of the cohort of De Kruijf et al. in combination with immune status of tumours. Determination of classical human leukocyte antigen (HLA) class I, HLA-E, HLA-G, natural killer cells and/or regulatory T cells additional to TSR showed to have an even stronger prognostic effect [16].

Dekker et al. investigated the prognostic value of the amount of stroma determined by visual eyeballing in 403 premenopausal node-negative breast cancer patients (cT1–3) [14]. These patients were selected from the perioperative chemotherapy trial (POP trial, 10854) [48]. This study supported the earlier finding of TSR as an independent prognostic parameter for disease-free survival (DFS) (HR 1.85, 95% CI 1.33–2.59, *P* < 0.001) in favour of stroma-low tumours, and borderline statistical significance for OS (HR 1.60, 95% CI 1.00–2.57, *P* = 0.050) [14].

Gujam et al. investigated patients with invasive carcinoma of no special type (NST) (T1–3, N0–>3, grade I-III,) and subsequently found a correlation between stroma-high tumours and a poor 15-year cancer-specific survival (CSS) (HR 2.12, 95% CI 1.37–3.29, *P* = 0.001) in multivariate survival analysis after visual TSR assessment of H&E slides of 361 patients [15].

Downey et al. dispute this finding in his work by analysing the stromal content with semi-automated point counting [46]. They found that high tumour–stroma content in 118 women with ER+ grade 1–3 invasive breast tumours was independently associated with a better overall survival (OS) and relapse-free survival (RFS) (95% CI 0.2–0.7, *P* = 0.008 and 95% CI 0.1–0.6, *P* = 0.006, respectively) [46].

Following on their first study, Downey and colleagues investigated the stromal content in 45 patients with inflammatory breast cancer, a rare and aggressive form of breast cancer, using the semi-automated point counting method [47, 49]. However, no statistical significant difference was observed for this series (OS *P* = 0.53, DFS *P* = 0.66) [47].

Roeke et al. (T1–3, N0–2, grade I–III general BC) validated, by visual TSR assessment, that a high stromal content was a prognostic factor for worse OS (HR 1.56, 95% CI 1.18–2.05, *P* = 0.002), distant metastasis-free survival (DMFS) (HR 1.52, 95% CI 1.12–2.06, *P* = 0.008) and RFS (HR 1.35, 95% CI 1.01–1.81, *P* = 0.046), in their study of 737 patients with primary operable invasive breast cancer [17]. Unlike the work of Downey et al., patients with ER + stroma-high tumours were associated with a worse OS (HR 1.43, 95% CI 1.04–1.99, *P* = 0.030) [17].

### Tumour–stroma ratio in triple-negative breast cancer

With respect to the applicability of TSR as a prognostic parameter in TNBC patients, a study has been performed by Moorman et al. in 2012. They analysed TSR in a retrospective cohort study consisting of triple-negative breast cancer patients (pT1–4, pN0–3 grade I–III) (*N* = 124) [13]. The amount of stroma was evaluated by visual eyeballing. Multivariate Cox regression analysis showed that the TSR was an independent prognostic factor for both RFP (HR 2.39, 95% CI 1.07–5.29, *P* = 0.033) and OS (HR 3.00, 95% CI 1.08–8.32, *P* = 0.034) in favour of stroma-low tumours. The 5-year RFP and OS for patients with stroma-low tumours compared to stroma-high tumours were 85% and 89% versus 45% and 65%, respectively [13].

A subgroup analysis for TNBC in the cohort of De Kruijf et al. of 82 TNBC patients supported the results of Moorman and colleagues that patients with stroma-high tumours had a significant shorter RFP (HR 2.92, 95% CI 1.36–6.32, *P* = 0.006) and OS (HR 1.87, 95% CI 1.07–3.26, *P* = 0.028) [12]. After 5 years of follow-up, 81% of the TNBC patients with stroma-low tumours were relapse free compared to 56% of patients with stroma-high tumours [12].

Among the 403 patients in the cohort of Dekker and colleagues, 69 patients were diagnosed with TNBC. Separate analyses of patients with stroma-high TNBC validated a 2.71 greater risk of developing a recurrence compared to patients with stroma-low TNBC (DFS; HR 2.71, 95% CI 1.11–6.61, *P* = 0.028) [14].

However, in the study of Gujam et al., the percentage of tumour–stroma was not found to be an independent prognostic factor for cancer-specific survival in 151 TNBC patients (*P* = 0.151) [15]. Likewise, Roeke et al. were not able to prove this correlation as well (*P* = 0.221) (Table 1) [17].

## Tumour–stroma ratio in other subgroups

De Kruijf et al., Gujam et al. and Roeke et al. described the role of TSR in other subgroups. The results of De Kruijf et al. showed an independent prognostic value of TSR in patients who received only local therapy (*P* < 0.001), only adjuvant chemotherapy (*P* = 0.038) and only adjuvant endocrine therapy (*P* = 0.024) [12]. The latter was confirmed by Roeke et al. (*P* = 0.001) [17]. The same results were seen in patients with a TN tumour who received only local therapy (*P* = 0.006).

In non-TNBC patients (*P* = 0.013), ER-positive patients (*P* = 0.030) and HER2-negative tumours, the TSR was also of independent prognostic value [12, 17]. This was not the case for ER- and PR-negative breast tumours [17]. The last subgroup in which TSR was evaluated and proved to be statistical significant was node-negative tumours (*P* = 0.002 and *P* = 0.003) [15, 17]. A summary of these results is presented in Table 2.

**Table 2:** The results of the multivariate Cox regression analyses on the prognostic value of TSR in different subgroups of breast tumours described in literature (data on main cohort of publication and TN tumours are presented in Table 1). Stroma-low is used as reference

Subgroup	De Kruijf et al. [12]				Gujam et al. [15]				Roeke et al. [17]			
	Recurrence-free period				Cancer-specific survival				Overall survival			
	*N* stroma-high (%)	HR	95% CI	*P*-value	*N* stroma-high (%)	HR	95% CI	*P*-value	*N* stroma-high (%)	HR	95% CI	*P*-value
Treatment												
Only local therapy (no systemic therapy)	244 (66)	2.06	1.42–2.97	< 0.001								
Only adjuvant chemotherapy	88 (68)	1.83	1.04–3.25	0.038								
Only adjuvant endocrine therapy	27 (29)	2.59	1.13–5.91	0.024						2.02	1.34–3.07	0.001
Only local therapy in TNBC		4.12	1.49–11.39	0.006								
Receptor status												
Non-TNBC		1.50	1.09–2.07	0.013								
ER-positive tumours										1.43	1.04–1.99	0.030
ER-negative and PR-negative tumours												No statistically significant difference (data not shown)
HER2-negative tumours												Results comparable with results of oestrogen receptor positive (data not shown)
Tumour stage												
Node-negative tumours					54 (26)	3.11	1.53–6.33	0.002		1.90	1.24–2.90	0.003

*ER* oestrogen receptor, *PR* progesterone receptor, *HER2* human epidermal growth factor receptor 2, *TNBC* triple-negative breast cancer. *HR* hazard ratio, *CI* confidence interval

## Discussion of current literature

Extensive research has been performed to determine prognostic biomarkers for patient prognosis. Molecular tests, as MammaPrint and Oncotype DX, have seemed to be valuable for the improvement of clinical decision making in early-stage breast cancer [7, 8]. These tests will possibly be endorsed into routine clinical practice in order to reduce the administration of adjuvant chemotherapy and prevent overtreatment [9]. However, the disadvantages of the aforementioned molecular testing are the relatively high cost and the far mostly unknown influence of tumour heterogeneity. More specifically, intermingled non-tumour tissue may have a profound influence on the test results [50].

TSR has shown to be of prognostic value in addition to the traditional prognostic markers which are implemented in standard clinical care, for example, TNM stage, receptor status and HER2 expression, in breast cancer with a robust inter-observer variability. In Supplementary Tables 1 and 2, the effect of TSR in addition to the most important traditional prognostic markers is shown for the entire study population and triple-negative tumours, respectively.

So far, seven studies regarding TSR have been performed in the field of breast cancer, of which five have shown a significant association between high tumour–stroma content and a poor prognosis [12–15, 17]. However, the results of both studies of Downey and colleagues were not in line with the other five [46, 47]. As Downey et al. have determined the TSR in both studies with semi-automated point counting instead of visual eyeballing and have utilised different cut-off values, it may be concluded that a standardised estimation of TSR is essential for a robust method, applicable for patient management. The method of determining TSR differed considerably, resulting in underestimating the heterogeneity [51]. In contrast with previous studies, where the final TSR category is based on the highest stroma rate in the sample, Downey and colleagues scored only an area of 9 mm^2^ at the edge of the tumour [10, 46, 51, 52].

Although the difference in results can possibly be attributed to this inconsistency, the different breast cancer subgroups regarding basic characteristics must be taken in consideration as well. The applicability in the subtypes, namely TNBC, ER+ and inflammatory breast cancer, may differ and subsequently the individual relevance of the TSR has to be determined in different breast cancer subgroups, as is previously performed by Roeke and colleagues [17]. For example, in lobular carcinomas, the question is raised on how to determine which part is tumour-induced stroma or supportive stroma. This should be further determined in larger cohorts. With respect to TNBC, five studies have investigated this subgroup, of which three studies have shown significant results [12–15, 17]. The results of these three studies are rather promising regarding the prognostic effect of TSR [12–14]. However, two other studies have not validated this prognostic effect despite the favourable results earlier showed. As mentioned by Roeke et al., this discordance could possibly be contributed to the relatively low amount of stroma-high tumours in the TNBC subgroup [17]. The similar reason could be the cause for the effect of TSR in TNBC patients in the study of Gujam et al. [15]. Another explanation could be that the histological type of TNBC plays a role.

Although different studies researched the prognostic value of TSR, little is known about the composition of the stroma. Even when using conventional light microscopy, vast differences in stromal morphology can be appreciated which are surely reflective of enormous differences in stromal functionality. Molecular analyses have identified multiple molecular markers that are associated with varying degrees of stromal activation [53–55]. These findings might allow us to distinguish activated, highly tumour-promoting stromal tissues from non-activated or only mildly active stromal tissues. Future studies investigating stromal activation might therefore solely focus on specific highly active subsets of stromal tissues as opposed to counting all stromal tissues equally, thereby further refining this parameter: For instance, by identifying PA28 as a marker of stromal activation in a previous publication [53].

Similarly, Ahn et al. investigated the stromal composition of breast cancer tissue. Besides TSR, the dominant histological stroma type (collagen, fibroblast or lymphocytes) offers additional prognostic information. 5- and 10-year RFS rates were most favourable in the lymphocytic stroma type, followed by the fibroblast and collagen type. The latter was associated with the most aggressive tumour and consequently poorest prognosis [56]. Interestingly, Ahn et al. observed a trend between TNBC and a predominantly lymphocytic stroma type, with 56.1% of the samples being classified as ‘lymphocytic’. Considering the fact that TNBC has a relatively poor prognosis, the observed trend between TNBC and a predominantly lymphocytic stroma type, with a favourable prognosis, is striking. Leon-Ferre and colleagues showed similar results in early-stage TNBC in which the presence of low tumour-infiltrating lymphocytes (TILs) contributes to a poor prognosis [57].

Considering the aforementioned generally promising prognostic effect in TNBC, this subgroup is the most obvious candidate for further exploration of TSR. Currently, adjuvant systemic chemotherapy is advocated for all patients that present with operable TNBC due to the aggressive nature of this tumour subgroup. Regarding TNBC, unlike other molecular subtypes, there is no FDA approved targeted therapy yet. Forasmuch as both the aggressive nature of the subtype as the devoid of therapeutic options, supplementary research is necessary. In order to develop curative therapeutics in TNBC, stromal targets have to be determined. Given the fact that TNBC predominantly consists of lymphocytic stroma, according to Ahn and colleagues, the possible target might lie within this stroma. The quantity of programmed death ligand 1 (PD-L1), expressed on tumour cells, could be prognostic as well. Tomioka et al. have shown that low TILs in combination with high PD-L1 expression predict an unfavorable prognosis. Within the abundant lymphocytic stroma in TNBC, PD-L1 could possibly operate as a target for therapeutic options [58]. Thus, in further research, in addition to a standardised estimation of TSR, the biology or quality of the stroma should be taken into account as well, in both general breast cancer and especially in TNBC patients in order to clarify the paradox and subsequently to lay a foundation regarding targeted therapy.

Lastly, it should be noted that although previous studies demonstrated prognostic value in the past, these studies have always been performed as part of retrospective studies by researchers and pathologists with a specific interest in stromal tissues. Breast cancer is a heterogeneous disease and for this reason additional larger retrospective studies could add valuable information about the prognostic value of TSR in specific subgroups as well. Moreover, no prospective feasibility studies have been performed and as such, it remains to be seen whether broad application of this parameter would lead to reproducible test results. Current research efforts in this direction are however ongoing.

## Conclusion

The current breast cancer prognostication schemes do not adequately predict patient prognosis. This leads to both over- and undertreatment with adjuvant chemotherapy. In order to better predict tumour biology and prevent unwarranted chemotherapy, additional prognostic parameters are necessary. TSR can be a valuable biomarker for determining patient prognosis. The scoring can be easily performed by the pathologist during routine pathological examination of H&E stained slides in less than a minute, with no additional costs, as it is a quick, simple method with a high reproducibility. The field of tumour–stroma provides promising perspectives, although standardisation of the methodology is desired. There is a trend towards high stromal content and a poor prognosis, being most applicable in TNBC. TSR in this case could be used to predict both disease progression and patient prognosis.

## Electronic supplementary material

Below is the link to the electronic supplementary material.

Supplementary material 1 (DOCX 38 KB)
