# Supplementary material for: The prognostic value of tumour–stroma ratio in primary breast cancer with special attention to triple-negative tumours: a review
Source: Breast Cancer Res Treat. 2018 Oct 9;173(1):55–64. doi: 10.1007/s10549-018-4987-4 (PMC6394568; doi:10.1007/s10549-018-4987-4)
Supplement: Supplementary file 1 — Supplementary material 1 (DOCX 38 KB) [file 10549_2018_4987_MOESM1_ESM.docx]

**Supplementary Table 1** Prognostic value of TSR in addition to traditional prognostic tumour characteristics in the main study cohort discussed in the literature calculated by univariate Cox- regression analyses.

| **Tumour characteristics** | **De Kruijf et al., 2011** | | | | | | **Dekker et al. 2013** | | | | | | **Gujam et al., 2014** | | | | | |
| --- | --- | --- | --- | --- | --- | --- | --- | --- | --- | --- | --- | --- | --- | --- | --- | --- | --- | --- |
|  | General breast cancer cohort | | | | | | General breast cancer cohort | | | | | | invasive carcinoma of no special type | | | | | |
|  | Recurrence-free period | | | | | | Disease-free survival | | | | | | Cancer-specific survival | | | | | |
|  | *Univariate* | | | *Multivariate* | | | *Univariate* | | | *Multivariate* | | | *Univariate* | | | *Multivariate* | | |
|  | HR | 95% CI | *P*- value | HR | 95% CI | *P*- value | HR | 95% CI | *P*- value | HR | 95% CI | *P*- value | HR | 95% CI | *P*- value | HR | 95% CI | *P*- value |
| Tumour size | 2.49 | 1.71-3.64 | <0.001 | 1.86 | 1.24-2.79 | 0.009 | 3.17 | 1.37-7.36 | 0.024 | 2.72 | 0.99-7.47 | 0.150 | 2.17 | 1.54-3.07 | <0.001 |  |  | 0.142 |
| Lymph node involvement | 3.06 | 2.38-3.95 | <0.001 | 2.66 | 2.03-3.49 | <0.001 |  |  |  |  |  |  | 1.97 | 1.51-2.56 | <0.001 | 1.97 | 1.46-2.66 | <0.001 |
| Tumour grade | 2.02 | 1.33-3.08 | 0.001 | 1.71 | 1.09-2.70 | 0.022 | 1.85 | 1.26-2.72 | 0.006 | 1.32 | 0.82-2.13 | 0.440 | 1.85 | 1.30-2.60 | <0.001 | 1.72 | 1.18-2.51 | 0.005 |
| Histological type | 1.24 | 0.83-1.85 | 0.291 |  |  |  |  |  |  |  |  |  |  |  |  |  |  |  |
| ER status | 1.05 | 0.81-1.36 | 0.725 |  |  |  | 0.87 | 0.60-1.26 | 0.454 |  |  |  | 0.52 | 0.34-0.79 | 0.002 |  |  | 0.240 |
| PR status | 0.96 | 0.74-1.24 | 0.744 |  |  |  | 0.83 | 0.60-1.16 | 0.275 |  |  |  | 0.44 | 0.32-0.82 | 0.006 |  |  | 0.184 |
| HER2 status | 1.21 | 0.78-1.88 | 0.401 |  |  |  | 1.27 | 0.83-1.97 | 0.275 |  |  |  | 1.44 | 0.88-2.35 | 0.145 |  |  |  |
| Ki-67 expression | 1.00 | 0.71-1.42 | 0.994 |  |  |  | 2.06 | 1.30-3.27 | 0.002 | 1.73 | 1.02-2.92 | 0.042 |  |  |  |  |  |  |
| Lymphovascular invasion |  |  |  |  |  |  |  |  |  |  |  |  | 2.07 | 1.39-3.09 | <0.001 |  |  | 0.864 |
| Angiogenesis |  |  |  |  |  |  | 1.21 | 0.81-1.80 | 0.349 |  |  |  |  |  |  |  |  |  |
| Tumour-stroma ratio | 1.62 | 1.23-2.13 | 0.001 | 1.97 | 1.47-2.64 | <0.001 | 1.69 | 1.23-2.31 | 0.001 | 1.85 | 1.33-2.59 | <0.001 | 1.89 | 1.26-2.82 | <0.001 | 2.12 | 1.37-3.29 | 0.001 |

The reference group used in the univariate and multivariate Cox regression analyses was different between the included studies. In this table only the traditional prognostic markers are shown. In the original papers more parameters were included in de multivariate analyses. Tumour size; pT1 versus pT3/4 (De Kruijf et al.), cT1 versus cT3 (Dekker et al.), ≤20mm versus >50mm (Gujam et al.) and ≤20mmversus >50mm (Roeke et al.). Lymph node involvement; pN negative versus pN positive (De Kruijf et al.), 0 versus >3 involved lymph nodes (Gujam et al.) and pN0 versus pN3 (Roeke et al.). Tumour grade; grade I versus grade III. Histological type; invasive carcinoma of no special type versus other (De Kruijf et al.) and invasive carcinoma of no special type versus lobular (Roeke et al.). Oestrogen receptor (ER) status; negative versus positive. Progesterone receptor (PR) status; negative versus positive. Human epidermal growth factor receptor 2 (HER2) status; no overexpression versus overexpression (De Kruijf et al.) and negative versus positive (Dekker et al. and Roeke et al.). Ki-67 expression; negative versus positive (De Kruijf et al.) and low versus high (Dekker et al.). Angiogenesis; low microvessel density versus high microvessel density. Lymphovascular invasion; no versus yes. Tumour-stroma ratio; stroma-low versus stroma-high. Abbreviations: HR = hazard ratio, CI = confidence interval

**CONTINUED Supplementary Table 1** Prognostic value of TSR in addition to traditional prognostic tumour characteristics in the main study cohort discussed in the literature calculated by univariate Cox- regression analyses.

| **Tumour characteristics** | **Downey et al., 2014** | | | | | | **Downey et al., 2015** | | | | | | **Roeke et al., 2017** | | | | | |
| --- | --- | --- | --- | --- | --- | --- | --- | --- | --- | --- | --- | --- | --- | --- | --- | --- | --- | --- |
|  | Oestrogen receptor positive breast cancer | | | | | | Only inflammatory breast cancer | | | | | | General breast cancer | | | | | |
|  | Relapse-free survival | | | | | | Disease-free survival | | | | | | Recurrence-free survival | | | | | |
|  | *Univariate* | | | *Multivariate* | | | *Univariate* | | | *Multivariate* | | | *Univariate* | | | *Multivariate* | | |
|  | HR | 95% CI | *P*- value | HR | 95% CI | *P*- value | HR | 95% CI | *P*- value | HR | 95% CI | *P*- value | HR | 95% CI | *P*- value | HR | 95% CI | *P*- value |
| Tumour size |  |  |  |  |  |  |  |  |  |  |  |  | 1.61 | 0.40-6.52 | 0.670 | 1.33 | 0.30-5.84 | 0.71 |
| Lymph node involvement |  |  |  |  |  |  |  |  |  |  |  |  | 4.41 | 2.68-7.26 | <0.001 | 4.58 | 2.51-8.36 | <0.001 |
| Tumour grade |  |  |  |  |  |  |  |  |  |  |  |  | 2.73 | 1.74-4.30 | <0.001 | 1.78 | 1.06-2.99 | 0.028 |
| Histological type |  |  |  |  |  |  |  |  |  |  |  |  | 0.97 | 0.61-1.55 | 0.910 |  |  |  |
| ER status |  |  |  |  |  |  |  |  |  |  |  |  | 0.53 | 0.38-0.74 | <0.001 | 0.82 | 0.52-1.28 | 0.375 |
| PR status |  |  |  |  |  |  |  |  |  |  |  |  | 0.59 | 0.44-0.79 | <0.001 | 0.78 | 0.54-1.12 | 0.183 |
| HER2 status |  |  |  |  |  |  |  |  |  |  |  |  | 1.09 | 0.77-1.54 | 0.633 | 1.04 | 0.73-1.48 | 0.819 |
| Ki-67 expression |  |  |  |  |  |  |  |  |  |  |  |  |  |  |  |  |  |  |
| Lymphovascular invasion |  |  |  |  |  |  |  |  |  |  |  |  |  |  |  |  |  |  |
| Angiogenesis |  |  |  |  |  |  |  |  |  |  |  |  |  |  |  |  |  |  |
| Tumour-stroma ratio |  | 0.4-2.8 | 0.001 |  |  |  |  |  | 0.66 |  |  |  | 1.26 | 0.95-1.67 | 0.113 | 1.35 | 1.01-1.81 | 0.046 |

**Supplementary Table 2** Prognostic value of TSR in addition to traditional prognostic tumour characteristics in triple negative breast cancer population described in the discussed literature calculated by univariate Cox regression analyses.

| **Tumour characteristics** | **De Kruijf et al., 2011** | | | | | | **Moorman et al., 2012** | | | | | | **Dekker et al., 2013** | | | | | |
| --- | --- | --- | --- | --- | --- | --- | --- | --- | --- | --- | --- | --- | --- | --- | --- | --- | --- | --- |
|  | Recurrence-free period | | | | | | Relapse- free period | | | | | | Disease-free survival | | | | | |
|  | *Univariate* | | | *Multivariate* | | | *Univariate* | | | *Multivariate* | | | *Univariate* | | | *Multivariate* | | |
|  | HR | 95% CI | *P*- value | HR | 95% CI | *P*- value | HR | 95% CI | *P*- value | HR | 95% CI | *P*- value | HR | 95% CI | *P*- value | HR | 95% CI | *P*- value |
| Tumour size | 3.53 | 1.03-12.08 | 0.131 |  |  |  |  |  |  |  |  |  | 10.17 | 2.29-45.11 | 0.009 | 6.19 | 1.04-36.96 | 0.075 |
| Lymph node involvement | 2.30 | 1.61-4.57 | 0.017 | 1.88 | 0.89-3.96 | 0.096 | 3.38 | 1.27-9.00 | 0.010 |  |  |  |  |  |  |  |  |  |
| Tumour grade | 1.72 | 0.80-3.69 | 0.163 |  |  |  |  |  |  |  |  |  | 0.84 | 0.12-6.22 | 0.478 |  |  |  |
| Histological type |  |  |  |  |  |  |  |  |  |  |  |  |  |  |  |  |  |  |
| Ki-67 expression | 0.70 | 0.31-1.61 | 0.403 |  |  |  |  |  |  |  |  |  | 2.44 | 0.33-17.91 | 0.382 |  |  |  |
| Lymphovascular invasion |  |  |  |  |  |  | 2.46 | 1.19-5.07 | 0.012 |  |  |  |  |  |  |  |  |  |
| Angiogenesis |  |  |  |  |  |  |  |  |  |  |  |  | 1.53 | 0.61-3.84 | 0.364 |  |  |  |
| Tumour-stroma ratio | 3.19 | 1.49-6.83 | 0.003 | 2.92 | 1.36-6.32 | 0.006 | 2.93 | 1.37-6.26 | 0.004 | 2.39 | 1.07-5.29 | 0.033 | 2.21 | 1.004-4.84 | 0.049 | 2.71 | 1.11-6.61 | 0.028 |

The reference group used in the univariate and multivariate Cox regression analyses was different between the included studies. In this table only traditional prognostic markers are shown. In the original papers more parameters were included in de multivariate analyses. Tumour size; pT1 versus pT3/4 (De Kruijf et al.), T1 versus T3 (Dekker et al). and ≤20mm versus >50mm (Gujam et al.). Lymph node involvement; pN negative versus pN positive (De Kruijf et al.), pN0 versus pN2/3 (Moorman et al.) and 0 versus >3 involved lymph nodes (Gujam et al.). Tumour grade; grade I/II versus grade III (De Kruijf et al.), grade I versus grade III (Gujam et al and Dekker et al.). Ki-67 expression; negative versus positive (De Kruijf et al.) and low versus high (Dekker et al.). Lymphovascular invasion; no versus yes. Angiogenesis; low microvessel density versus high microvessel density. Tumour-stroma ratio; stroma-low versus stroma-high. Abbreviations: HR = hazard ratio, CI = confidence interval

| **Tumour characteristics** | **Gujam et al., 2014** | | | | | | **Roeke et al., 2017** | | | | | |
| --- | --- | --- | --- | --- | --- | --- | --- | --- | --- | --- | --- | --- |
|  | Cancer-specific survival | | | | | | Overall survival | | | | | |
|  | *Univariate* | | | *Multivariate* | | | *Univariate* | | | *Multivariate* | | |
|  | HR | 95% CI | *P*- value | HR | 95% CI | *P*- value | HR | 95% CI | *P*- value | HR | 95% CI | *P*- value |
| Tumour size | 3.10 | 1.91-5.04 | <0.001 | 2.53 | 1.52-4.21 | <0.001 |  |  |  |  |  |  |
| Lymph node involvement | 1.91 | 1.34-2.71 | <0.001 | 1.64 | 1.15-2.34 | 0.007 |  |  |  |  |  |  |
| Tumour grade | 0.97 | 0.54-1.74 | 0.916 |  |  |  |  |  |  |  |  |  |
| Histological type |  |  |  |  |  |  |  |  |  |  |  |  |
| Ki-67 expression |  |  |  |  |  |  |  |  |  |  |  |  |
| Lymphovascular invasion | 2.11 | 1.20-3.70 | 0.009 |  |  |  |  |  |  |  |  |  |
| Angiogenesis |  |  |  |  |  |  |  |  |  |  |  |  |
| Tumour-stroma ratio | 1.06 | 1.03-1.12 | 0.035 |  |  | 0.151 |  |  | No statistical significant difference (data not shown) |  |  |  |

**CONTINUED Supplementary Table 2** Prognostic value of TSR in addition to traditional prognostic tumour characteristics in triple negative breast cancer population described in the discussed literature calculated by univariate Cox regression analyses

| **Subgroup** | **De Kruijf et al., 2011** | | | | **Gujam et al., 2014** | | | | **Roeke et al. 2017** | | | |
| --- | --- | --- | --- | --- | --- | --- | --- | --- | --- | --- | --- | --- |
|  | Recurrence free period | | | | Cancer-specific survival | | | | Overall survival | | | |
|  | *N*  stroma-high (%) | HR | 95% CI | *P*- value | *N*  stroma-high (%) | HR | 95% CI | *P*- value | *N*  stroma-high (%) | HR | 95% CI | *P*- value |
| **TREATMENT** | | | | | | | | | | | | |
| Only local therapy  (no systemic therapy) | 244 (66) | 2.06 | 1.42-2.97 | <0.001 |  |  |  |  |  |  |  |  |
| Only adjuvant chemotherapy | 88 (68) | 1.83 | 1.04-3.25 | 0.038 |  |  |  |  |  |  |  |  |
| Only adjuvant endocrine therapy | 27 (29) | 2.59 | 1.13-5.91 | 0.024 |  |  |  |  |  | 2.02 | 1.34-3.07 | 0.001 |
| Only local therapy in TNBC |  | 4.12 | 1.49-11.39 | 0.006 |  |  |  |  |  |  |  |  |
| **RECEPTOR STATUS** | | | | | | | | | | | | |
| Non- TNBC |  | 1.50 | 1.09-2.07 | 0.013 |  |  |  |  |  |  |  |  |
| ER positive tumours |  |  |  |  |  |  |  |  |  | 1.43 | 1.04-1.99 | 0.030 |
| ER negative and PR negative tumours |  |  |  |  |  |  |  |  |  |  |  | No statistically significant difference (data not shown) |
| HER2-negative tumours |  |  |  |  |  |  |  |  |  |  |  | Results comparable with results of estrogen receptor positive (data not shown) |
| **TUMOUR STAGE** | | | | | | | | | | | | |
| Node- negative tumours |  |  |  |  | 54 (26) | 3.11 | 1.53-6.33 | 0.002 |  | 1.90 | 1.24-2.90 | 0.003 |

**Supplementary Table 3** The results of the multivariate Cox regression analyses on the prognostic value of TSR in different subgroups of breast tumours described in literature (data on main cohort of publication and TN tumours are presented in Table 1).

Stroma-low is used as reference. Abbreviations: ER = oestrogen receptor, PR = progesterone receptor, HER2 = human epidermal growth factor receptor 2, TNBC = triple negative breast cancer. Abbreviations: HR = hazard ratio, CI = confidence interval
